# A Review on the Use of Artificial Intelligence in Fracture Detection

**DOI:** 10.7759/cureus.58364

**Published:** 2024-04-16

**Authors:** Aayushi Bhatnagar, Aditya L Kekatpure, Vivek R Velagala, Aashay Kekatpure

**Affiliations:** 1 Medicine, Jawaharlal Nehru Medical College, Datta Meghe Institute of Higher Education and Research, Wardha, IND; 2 Orthopedic Surgery, Jawaharlal Nehru Medical College, Datta Meghe Institute of Higher Education and Research, Wardha, IND; 3 Orthopedic Surgery, Narendra Kumar Prasadrao Salve Institute of Medical Sciences and Research, Nagpur, IND

**Keywords:** natural language processing, orthopedic traumatology, radio-diagnosis, recurrent neural networks, convolutional neural networks, deep learning, machine learning, artificial intelligence

## Abstract

Artificial intelligence (AI) simulates intelligent behavior using computers with minimum human intervention. Recent advances in AI, especially deep learning, have made significant progress in perceptual operations, enabling computers to convey and comprehend complicated input more accurately. Worldwide, fractures affect people of all ages and in all regions of the planet. One of the most prevalent causes of inaccurate diagnosis and medical lawsuits is overlooked fractures on radiographs taken in the emergency room, which can range from 2% to 9%. The workforce will soon be under a great deal of strain due to the growing demand for fracture detection on multiple imaging modalities. A dearth of radiologists worsens this rise in demand as a result of a delay in hiring and a significant percentage of radiologists close to retirement. Additionally, the process of interpreting diagnostic images can sometimes be challenging and tedious. Integrating orthopedic radio-diagnosis with AI presents a promising solution to these problems. There has recently been a noticeable rise in the application of deep learning techniques, namely convolutional neural networks (CNNs), in medical imaging. In the field of orthopedic trauma, CNNs are being documented to operate at the proficiency of expert orthopedic surgeons and radiologists in the identification and categorization of fractures. CNNs can analyze vast amounts of data at a rate that surpasses that of human observations. In this review, we discuss the use of deep learning methods in fracture detection and classification, the integration of AI with various imaging modalities, and the benefits and disadvantages of integrating AI with radio-diagnostics.

## Introduction and background

Artificial intelligence (AI) is a relatively new and upcoming field of computer science that uses algorithms to mimic human intelligence and assist or augment humans in performing specific tasks [1]. These algorithms determine the probability of several different outcomes of new data from patterns derived from an extensive collection of data [2]. The term “machine learning” is used to describe the process in AI that allows these algorithms to derive patterns from data so as to enhance their operation without depending on definite programming. “Deep learning” refers to the use of several layers of computing to arrive at a higher level of information in response to any input.

In the medical field, we have primarily used AI to arrive at image-based diagnoses and determine the probable outcomes following a treatment modality. There is much scope in the use of AI in radiological orthopedics, particularly in the detection of fractures, substituting as a service for screening fractures, assisting in decision-making, and as a second reader support for radiologists [3]. Fractures are a widespread condition that affects all age groups, and their diagnosis requires vigilance and accuracy to decide the patient's prognosis. Traditionally, this task relied on the expertise of trained radiologists who took to analyzing radiographs to detect and classify fractures. This process can be tedious, arbitrary, and vulnerable to human error. This is especially the case when dealing with subtle or complex fractures that can be easily missed by the human eye [4]. Human and environmental variables, such as inexperienced clinicians, weariness, interruptions, suboptimal viewing circumstances, and time constraints, can all play a role in errors of radiological interpretation. The continuous and uninterrupted assessment of radiographic images by computers could prove a great help to the field of diagnostic radiology [5].

With the advent of AI, there are possibilities for making more accurate and timely diagnoses with automated detection, localization, and classification through its ability to uncover insightful patterns from extensive data collections. This can play to the benefit of the treating doctor in making quick and efficient decisions in managing the case, assessing the severity of the fracture, and improving the patient outcome. It can also help in eliminating diagnostic variability between multiple observers [6]. 

**Figure 1:**
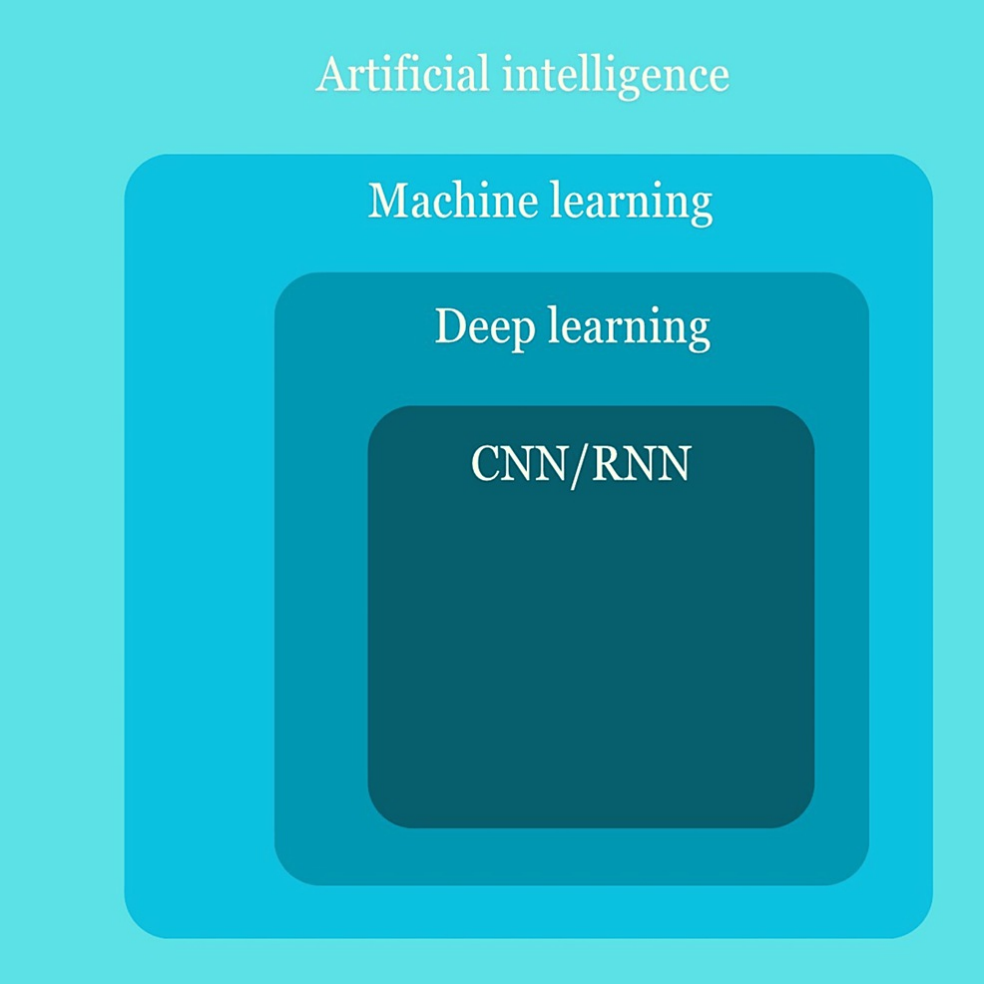
Diagrammatic representation of the subsets of AI focused on in this article AI, artificial intelligence The figure is the author’s own creation.

AI's deep learning subfield has revolutionized information technology (IT) by demonstrating significant potential in applications involving imaging in medicine. Convolutional neural networks (CNNs), which mimic human visual cortex neurons, and recurrent neural networks (RNNs), two types of deep learning models, may readily recognize patterns of fractures by acquiring hierarchical representations based on imagery. Performance optimization and increasing accuracy of fracture detection can be achieved by training these models using vast, annotated datasets [7]. The subsets of AI relevant to orthopedic radio-diagnostics are depicted in Figure 1. 

## Review

Search methodology

**Figure 2:**
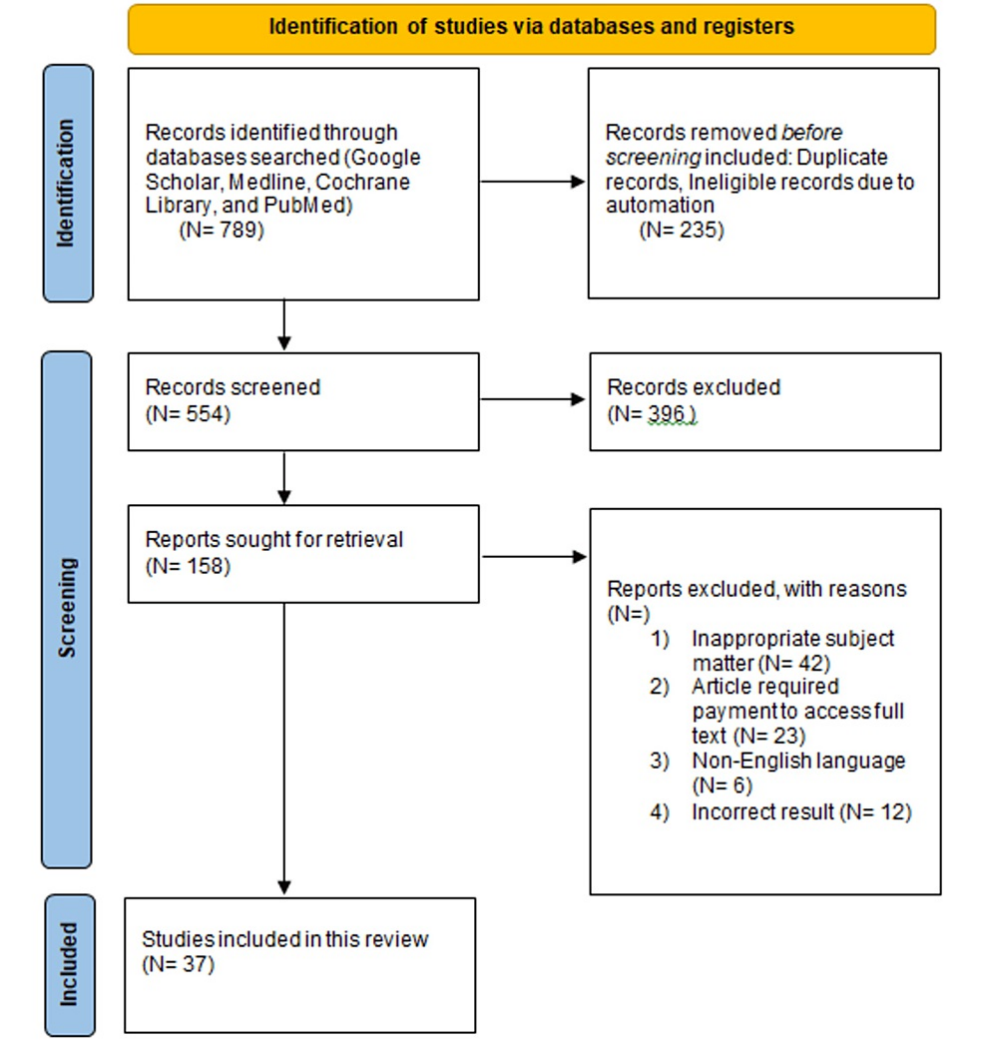
PRISMA flow diagram for inclusion and exclusion criteria PRISMA: Preferred Reporting Items for Systematic Review and Meta-Analyses

The researchers systematically investigated and compared the use of various AI techniques in orthopedic radio-diagnostics for this review paper. All research publications, including systematic reviews, experimental investigations of current fracture detection tests, and randomized and non-randomized clinical trials, were included. The researchers also considered additional methods, such as combining AI with other imaging modalities, and using natural language processing to gather data from patient records and published medical literature, in order to expedite and enhance the process of fracture detection and classification. Furthermore, the researchers conducted a comprehensive literature search through a variety of academic journals and search engines, including Google Scholar, Medline, Cochrane Library, and PubMed, using keywords such as "convolutional neural networks," "recurrent neural networks," "deep learning methodology," "ground truth," and "long short-term memory networks." The selection criteria employed in this study are as follows (Figure 2): (1) AI, (2) radio-diagnostics, (3) orthopedic traumatology, (4) deep learning methods, (5) CNNs, (6) RNNs, (7) natural language processing, and (8) English language. The following conditions were excluded: (1) inappropriate topic matter, (2) technical issue, (3) article required payment, and (4) non-English language. Figure 1 shows a PRISMA flow diagram for the search strategy.

Use of deep learning model in automated detection and localization of fractures

The use of deep learning models in fracture detection and classification has been on the rise. It has been an area of much speculation and has shown much promise. Utilizing their capacity to extract intricate patterns and characteristics from large databases, deep learning models have become practical tools for increasing the precision and effectiveness of fracture detection. Integrating these models with attention mechanisms and transfer learning has also led to further advancements in fracture detection. By using attention processes, the model may concentrate on essential areas or details in a picture, improving its capacity for discrimination. An attention-guided structure was created to recognize wrist fractures, using attention maps to emphasize key areas in the X-ray scans. When weighed against conventional CNN systems, the attention-guided model performed better, offering more precise fracture location and identification [8]. Fracture detection may also be done using transfer learning, a method that makes use of models that have already undergone training on massive datasets. In order to fit the goal of fracture detection and classification, these pre-trained models are refined using datasets relevant to fractures. Despite having relatively little data, this method improves adaptability and efficiency. High accuracy is achieved when using transfer learning with pre-trained models to identify fractured hips in X-ray images, illustrating the promise of transfer learning in fracture diagnosis [9].

Convolutional Neural Networks

These are a machine deep learning approach and have become increasingly popular in radio-diagnostics in the last few years. CNNs acquire identifying characteristics based on the pixel information of large image datasets to determine the diagnosis. Deep learning CNNs have become capable of functioning at levels comparable to humans in non-artificial domains, including face detection, handwriting identification, and natural-world picture categorization. This is made possible by ongoing advancements in CNN designs combined with a geometric rise in the processing capacity of the equipment. Early research using deep-learning CNNs for diagnosis in the field of medicine has demonstrated potential in classifying masses in mammograms, classifying pulmonary tuberculosis on chest X-rays, determining skeletal age, and classifying diabetic retinopathy. Research has also demonstrated the viability of CNNs for radiological detection of fractures [5]. This is further elaborated in Table 1 by the results of some studies [10-18].

**Table 1:** Findings of some studies done in the past seven years demonstrating the successful integration of CNNs in fracture detection and classification CT, computed tomography; CNN, convolutional neural network; R-CNN, region-based convolutional neural network; VGG, visual geometry group; AUC, area under the receiver operating characteristic curve; SURF, speeded-up robust feature

S.No.	Author	Year of study	Modality	Joint/bone under investigation	Description	Result
1.	Olczak et al. [10]	2017	X-ray	Hand, wrist, and ankle joints	Five freely accessible deep learning networks were selected to determine fractures in 256000 images of wrist, hand, and ankle radiographs. The best-performing system was compared to a gold standard for fractures. When examined under identical settings, the degree of precision of the best network was comparable to that of two senior orthopedic doctors. Notably, the most frequent reason for mistakes could have been unclear or missing information in the image.	Accuracy: 83%
2.	Nicolaes et al. [11]	2023	CT	Vertebrae	The algorithm effectively identified fractures of the vertebrae in the cohort of men and women aged ≥50, based on medical data, which retrospectively picked CT images of the abdomen, chest, and thoracic/lumbar spine.	Accuracy: 93%; sensitivity: 94%; specificity: 93%
3.	Wang et al. [12]	2022	CT	Mandible	Three skilled maxillofacial surgeons served as the standard, classifying and annotating the CT scans of 686 individuals with mandibular fractures. Using several CT images, an algorithm involving two CNNs was developed, verified, and examined. The algorithm's diagnostic efficacy was assessed by contrasting its results with the benchmark.	Accuracy: 90%; AUC=0.956
4.	Yang et al. [13]	2022	X-ray	Scaphoid	A two-step CNN was suggested in this investigation to identify scaphoid fractures. Utilizing the faster R-CNN network, scaphoid bone is first isolated from the radiograph. In the subsequent phase, the feature pyramid network and the convolutional block attention module are used to create the identification and categorization algorithms for scaphoid fractures, with the ResNet model serving as the foundation for obtaining features. The suggested approach's effectiveness was assessed using a range of measures, including recall, precision, sensitivity, specificity, accuracy, and AUC. The findings demonstrated that this technique offered useful benchmarks for scaphoid fracture measurement.	Accuracy: 99.70%; AUC: 0.920
5.	Prijs et al. [14]	2023	X-ray	Ankle	The Mask R-CNN model was used for this study to identify and classify ankle fractures. Radiographs that had been labeled and annotated were used for the conditioning of the CNN model. The ground truth label was determined by three expert trauma surgeons with fellowships in trauma care. The efficacy of the categorization was evaluated using diagnostic precision and AUC.	Accuracy: 89%; sensitivity: 89%; specificity: 96%.
6.	Oka et al. [15]	2021	X-rays	Radius and ulna	The CNN model used in this study is VGG16, which is a trained model for the identification of images. To locate the fractures in standard radiographs, it was changed into a network that included two output layers. While anteroposterior pictures were used to identify a styloid process fracture, both lateral and anteroposterior radiographs of the same patient were used to diagnose a distal radius fracture. The AI was assessed regarding diagnostic specificity, sensitivity, and accuracy.	Accuracy for distal radius fractures: 98.0±1.6%; AUC: 0.991; accuracy for fractures of the styloid process of the ulna: 91.1±2.5%; AUC: 0.956
7.	Pranata et al. [16]	2020	CT	Calcaneum	ResNet and VGG, two varieties of CNN methods with varying network levels, were assessed and contrasted for their ability to classify CT images into fracture and non-fracture groups according to coronal, sagittal, and transverse views. The methodology for detecting bone fractures included contour tracing, accurate border recognition, and fracture region comparison through the SURF approach.	Accuracy: 0.793; specificity: 0.729; sensitivity: 0.829
8.	Warin et al. [17]	2022	X-rays	Mandible	Retrospective access of 855 mandibular radiographs from 2016 to 2020 was attained from the local trauma facility. DenseNet-169 and ResNet-50, CNN-based algorithms for classification, were created to recognize fractures in the radiographs. An evaluation of the results was conducted using a test set in conjunction with comparisons with oral and maxillofacial surgery specialists on a subset of one hundred images. The models' performance in binary classification produced encouraging outcomes.	Sensitivity: 100%; specificity: 100%; AUC: 90%
9.	Chung SW et al. [18]	2018	X-rays	Proximal humerus	The purpose of the research was to assess CNN algorithms' performance in identifying and categorizing proximal humerus fractures based on 1891 plain AP shoulder X-rays. In contrast to the human participants, the CNN performed better than orthopedic surgeons and general practitioners, on par with shoulder-specific orthopedists, and ruled that AI can reliably identify and categorize proximal humerus fractures on standard AP X-rays of the shoulder.	Accuracy: 65-86%; AUC: 0.90-0.98

Recurrent Neural Networks

In fracture evaluation, RNNs, specifically, long short-term memory (LSTM) networks, have been implemented to make use of spatial data as well as serial interconnections. RNNs work effectively in jobs where informational background and sequence are important considerations. They can be applied for the accumulation of the extracted traits and for coming to the final diagnosis [19]. In a study conducted by Jasim K et al. on the identification and categorization of spinal column injuries, employing an osteoporotic vertebral fractures (OVF) database, the use of a deep RNN was compared against other existing models for injury classification. Injury identification was carried out using a deep CNN. After the presence and localization of the same on the vertebral column were determined, the suggested deep RNN was used to classify the level of the injury into normal, wedge, biconcavity, and crush. If there was no damage, the procedure was ended. The accuracy, sensitivity, and specificity of the suggested model were assessed, and the results showed that the deep RNN approach in question outperformed the other methods used to classify injuries. These values were 0.895, 0.871, and 0.933, respectively [20]. RNNs have also been used in the analysis of sequentially ordered clinical information, for instance, the time-series information gathered from sensors worn by patients or from medical files, for the purpose of fracture identification. An LSTM-based model that uses accelerometer readings to identify stress fractures successfully did so with excellent precision by capturing temporal trends in the data [21].

Combination of Convolutional Neural Network/Recurrent Neural Network

In terms of obtaining context-sensitive data and features of an image, the integration of CNNs and RNNs has demonstrated encouraging outcomes because it allows for the incorporation of both spatial and temporal data. An RNN/CNN hybrid was suggested in research conducted by Tomita et al. on the automated diagnosis of OVF on computed tomography (CT) images. Radiological characteristics were extracted from every slice of the scan using a CNN approach. Using an LSTM network, the features retrieved were run through a feature aggregating component to get the most accurate diagnosis for the entire scan. The outcomes were assessed against the results of diagnosis from practicing expert radiologists in actual clinical settings. An accuracy of 89.2% was attained by using this proposed method, combining both modalities [19]. Through the utilization of CNNs' capabilities in analyzing images and RNNs' advantages in serial computation of information, these hybrid models improve the accuracy of diagnostics and offer a thorough comprehension of fractures. Using their combined structures in fracture detection shows how these models may enhance medical investigations and choice-making regarding precision, effectiveness, and speed. These artificial neural network designs' capacity for fracture detection will be further improved by ongoing developments and the incorporation of more AI methods [22].

Use of natural language processing in fracture detection

Automatic techniques for searching medical information have been developed as a result of the increasing adoption of digital radiography and computerized health records, together with advancements in information technologies [23,24]. In recent years, the purpose of enhancing the acquisition and comprehension of pertinent data from reports on patients, radiological notes, and medical texts has led to a rise in demand for NLP approaches in fracture detection. Natural language processing (NLP) makes it possible to process and evaluate unstructured textual data automatically, which makes it easier to obtain information, classify it, and improve decision-making while diagnosing fractures [25]. NLP also provides the additional benefit of enhancing search performance through automatic machine-learning techniques [26]. NLP-based programs have been tested and used in an exploratory capacity to detect fractures, support radiology diagnoses, and determine medically meaningful changes. Conventional methods depend on the laborious and error-prone human assessment and transcription of radiological records. Information related to fracture type, its site, and any corresponding features may be automatically identified and extracted from free-text documents through NLP methods like named entity recognition and relationship extraction [27]. Studies have shown that NLP-based programs, such as the X-Ray Artificial Intelligence Tool (XRAIT), may identify individuals with previous fractures who are susceptible to osteoporosis three times more frequently. One theory regarding this software's high specificity and accuracy is that it uses a rule-based execution approach founded on specialist expertise [28]. Wang et al., in a control cohort conducted in 2019, demonstrated a sensitivity of 93% and specificity of 100% in the automated recognition of fractures at certain specific skeletal sites, which were related to osteoporosis using a rule-based NLP program [29].

Combining AI with other imaging modalities

AI's ability to work with different radiological modalities, including CT and magnetic resonance imaging (MRI), has created novel possibilities for precise and thorough fracture identification. Through the utilization of machine learning algorithms in conjunction with the distinct advantages associated with every technique, researchers have achieved noteworthy progress in fracture diagnosis, characterization, and therapy prescription. A more thorough assessment of fractures is made possible by the detailed cross-sectional pictures that CT imaging gives, especially for complicated fractures. AI systems have been created to diagnose fractures by analyzing CT scans. On thoracic CT scans, deep learning models can reliably identify and classify broken ribs, substantially minimizing the time needed for identification and lowering the likelihood of overlooked and incorrect diagnoses. It is possible to increase the accuracy of rib fracture diagnosis in chest CT scans with CNN methods [30].

Yet another helpful imaging technique for diagnosing fractures is MRI, which offers superior soft tissue perception and aids in evaluating soft tissue injuries. Interfacing AI with MRI has demonstrated the potential to enhance fracture characterization and identification. This study demonstrated how an approach developed using machine learning techniques could adequately determine the likelihood of a scaphoid fracture on an MRI based on factors such as gender, age, ulnar deformity, and scaphoid pain. The technique decreased the amount of patients having to get advanced scanning by a third, with a minuscule chance of failing to identify a fracture [31]. The conjunction of AI with different imaging techniques presents potential for digital surgery preparation and simulation. Surgeons can improve operative methods, anticipate fracture reduction, and assess the durability of stabilization by merging artificial neural networks with pre-operative scans. While the benefits of merging AI with other imaging modalities are plentiful, there are also concerns such as technical complexity, the need for scanning technique uniformity, and information compatibility. Furthermore, research is still being done to create strong AI models that can process multifaceted data as well as offer trustworthy fracture evaluation [32].

Applications in clinical practice - potential benefits and pitfalls

The previously cited studies of deep learning integration in radiology demonstrate the prospective advantages of developing and implementing machine learning algorithms in routine practice for both fracture description and identification. AI has the ability to supplement current radiological methods in a way that might increase the speed and precision of diagnosis while reducing the demand for professionals by enabling radiologists to focus on less time-consuming duties [7]. When weighed against experienced medical professionals, machine learning algorithms can serve as a trustworthy secondary opinion, increasing overall accuracy and notably lowering the number of overlooked fractures [33]. Machine learning techniques can also help find inconspicuous or complicated fractures that would be difficult to see with the human eye. Algorithms powered by AI can help in fracture classification as well as detection, as errors in classification may result in a substantial effect on planning the management. AI-based classification systems have the potential for standardizing fracture analysis, producing findings that are accurate and uniform. This lowers the possibility of incorrect categorization and guarantees the use of successful therapies that enhance outcomes for patients. Laborious operations may be mechanized by utilizing AI algorithms, freeing radiologists to concentrate on more complicated analyses, and opening up possibilities for quicker and simplified diagnostic operations. Moreover, it offers an opportunity to utilize medical assets better and save costs. AI may result in significant reductions in expenses and effective distribution of resources in the orthopedic diagnostic department by optimizing the diagnostic procedure, accelerating the evaluation and reporting process, decreasing needless radiological investigations, and improving efficiency [34].

Pitfalls

AI in fracture diagnosis brings up significant ethical and legal issues that require serious examination. The increasing prevalence of machine learning methods in medical environments necessitates careful consideration of ethical considerations, patient rights, anonymity, and confidentiality, as well as compliance with applicable legislation. Because a recommendation by an AI model lacks an operator interface, is not readily apparent, and cannot be challenged, a doctor may be hesitant to utilize it. Furthermore, it is still up for debate as to who might be deemed accountable if the algorithm makes a mistake and does harm, underscoring the necessity of having the right laws in place [2]. For the purpose of training the corresponding AI systems, the majority of studies employ databases containing ground truth labels derived from reports from radiologists. These might contain some inherent inaccuracies and incorrect interpretations due to human error. Improved and more detailed ground truth labeling might help us create AI systems that are more exact [35]. Furthermore, these AI algorithms could be able to identify the fracture, but they might not be able to identify which fractures, for example, might contain a bone tumor [36]. On the other hand, while reviewing radiographs of fractures, an orthopedic surgeon or radiologist is likely to identify other pertinent pieces of evidence. In addition, doctors possess the ability to integrate unbiased criteria and patient preferences into meticulous medical decision-making [37]. Table 2 depicts the summary of all the studies included in the article.

**Table 2:** Summary of studies included in the review CNN, convolutional neural networks; NLP, natural language processing; XRAIT, X-Ray Artificial Intelligence Tool; IT, information technology; AI, artificial intelligence; CT, computed tomography

S.No	Authors	Year	Summary
1.	Kuo RYL, Harrison C, Curran T-A, et al. [1]	2022	AI and clinicians had comparable reported diagnostic performance in fracture detection, suggesting that AI technology holds promise as a diagnostic adjunct in future clinical practice.
2.	Langerhuizen DWG, Janssen SJ, Mallee WH, et al. [2]	2019	Preliminary experience with fracture detection and classification using AI shows promising performance, and AI may enhance processing and communicating probabilistic tasks in medicine, including orthopedic surgery.
3.	McKinney SM, Sieniek M, Godbole V. et al. [3]	2020	A robust assessment of the AI system paves the way for clinical trials to improve the accuracy and efficiency of breast cancer screening and using a combination of AI and human inputs could help to improve screening efficiency.
4.	Chan HP, Samala RK, Hadjiiski LM, Zhou C [4]	2020	Deep learning, particularly through the use of hierarchical feature representations learned from data, has significantly advanced the interpretation of medical images, leading to improved identification, classification, and quantification of patterns in various medical applications such as image registration, anatomical/cell structures detection, tissue segmentation, and computer-aided disease diagnosis or prognosis.
5.	Thian YL, Li Y, Jagmohan P, Sia D, Chan VEY, Tan RT [5]	2019	The ability of an object detection CNN to detect and localize radius and ulna fractures on wrist radiographs with high sensitivity and specificity was demonstrated.
6.	Cui Y, Zhu J, Duan Z, Liao Z, Wang S, Liu W [6]	2022	AI can make images of the spine more useful to patients and doctors by improving image quality, imaging efficiency, and diagnostic accuracy through the convergence of imaging, AI, and radiomic techniques.
7.	Kalmet PHS, Sanduleanu S, Primakov S, et al. [7]	2020	The ways in which deep learning until now has been applied to fracture detection on radiographs and CT examinations are described and what value deep learning offers to this field is discussed; and future directions of this technology are commented on.
8.	Wang X, Peng Y, Lu L, Lu Z, Bagheri M, Summers RM [8]	2017	In this article, a radiomics-guided transformer is proposed to fuse global image information with local radiomics-guided auxiliary information to provide accurate cardiopulmonary pathology localization and classification without any bounding box annotations.
9.	Smets J, Shevroja E, Hügle T, Leslie WD, Hans D [9]	2021	In this article, the authors present a qualitative review of osteoporosis management using machine learning techniques in complex data environments where the human capacity to identify high-dimensional relationships is limited, notwithstanding technical and clinical concerns regarding the application of machine learning methods.
10.	Olczak J, Fahlberg N, Maki A, et al. [10]	2017	This study supports the use of orthopedic radiographs of AI, which can perform at a human level, while current implementation lacks important features that surgeons require.
11.	Nicolaes J, Liu Y, Zhao Y, et al. [11]	2023	In order to identify scans with vertebral fractures, the CNN method achieved a sensitivity of 94% and specificity of 93%. The method may help clinicians identify vertebral fractures early in conventional CT images of the abdomen and chest, according to the findings of the external validation.
12.	Wang X, Xu Z, Tong Y, et al. [12]	2022	In this paper, the authors evaluated the performance of CNN-based models for the detection and classification of maxillofacial fractures in CT bone window images. The study concluded that CNNs were equally dependable and accurate in identifying and categorizing mandibular fractures on CT scans. Medical professionals will benefit from the expertise provided by the computer program for automatic identification and categorization of maxillofacial fractures, which will also help to increase diagnosing performance.
13.	Yang TH, Horng MH, Li RS, Sun YN [13]	2022	A two-stage CNN is proposed to detect scaphoid fractures using the faster R-CNN network and uses the ResNet model as the backbone for feature extraction to develop the detection and classification models for scaphoids fractures.
14.	Prijs J, Liao Z, To MS, et al. [14]	2023	In this paper, the mask R-CNN was used for the segmentation of fracture lines on ankle radiographs, and a mean accuracy of 0.65 (SD±0.16) was observed.
15.	Oka K, Shiode R, Yoshii Y, Tanaka H, Iwahashi T, Murase T [15]	2021	In this article, the authors developed an AI system capable of diagnosing distal radius fractures with high accuracy even when learning with relatively small data by learning to use bi-planar X-ray images.
16.	Pranata YD, Wang KC, Wang JC, Idram I, Lai JY, Liu JW, Hsieh IH [16]	2019	A computer-aided method for calcaneal fracture detection achieves a faster and more detailed observation and attains a high precision rate of 86%, with a fast computational performance of 133 frames per second, used to analyze the severity of injury to the calcaneus.
17.	Warin K, Limprasert W, Suebnukarn S, Inglam S, Jantana P, Vicharueang S [17]	2022	In this article, a two-stage deep learning framework was used to detect mandibular trauma and fractures in panoramic radiographs, and a comparison was made between the accuracy, specificity, and sensitivity of AI and general dentists.
18.	Chung SW, Han SS, Lee JW, et al. [18]	2018	The CNN showed superior performance to that of general physicians and orthopedists, similar performance to orthopedists specialized in the shoulder, and the superior performance of the CNN was more marked in complex 3- and 4-part fractures.
19.	Tomita N, Cheung YY, Hassanpour S [19]	2018	In this paper, an end-to-end pipeline for the detection of osteoporotic compression fractures of the vertebral body in CT images is presented. However, the method is limited to a single vertebra.
20.	K MJ, Brindha T [20]	2021	The research paper proposes a model using optimized RNNs for spinal cord injury classification and level detection, achieving high accuracy, sensitivity, and specificity in experimentation. The experimental results show that the proposed deep RNN model is better than the existing models in terms of accuracy, sensitivity, and specificity.
21.	Wang Y, Oyen D, Guo W (Grace), et al. [21]	2021	A deep learning model is proposed to predict the entire sequence of maximum internal stress based on fracture propagation and the initial stress data, using the temporal independent CNN and the bidirectional LSTM, to reduce computational cost while preserving accuracy.
22.	Lex JR, Di Michele J, Koucheki R, Pincus D, Whyne C, Ravi B [22]	2023	In this paper, a systematic review and meta-analysis of 39 studies identified similar error rates of hip fracture diagnosis between AI models and expert clinicians and suggested that there was minimal advantage of machine learning models over traditional regression techniques for postoperative outcome prediction.
23.	Hassanpour S, Bay G, Langlotz CP [23]	2017	A natural language processing method to automatically extract clinical findings in radiology reports and characterize their level of change and significance according to a radiology-specific information model using a combination of machine learning and rule-based approaches.
24.	Demner-Fushman D, Chapman WW, McDonald CJ [24]	2009	Natural language processing is utilized in clinical decision support systems to extract and process unstructured clinical texts, enabling the development of predictive models for disease diagnosis. In this paper, a module for the extraction and pretreatment of patients' electronic medical record data was developed, which can be used for preliminary assessment of patient health status, as well as integrated into existing medical decision support systems.
25.	Névéol A, Zweigenbaum P [25]	2017	The paper discusses recent advancements in clinical NLP, highlighting tools for concept recognition, co-reference resolution, and temporal analysis, crucial for processing biomedical language data. The five clinical NLP best papers provide a contribution that ranges from emerging original foundational methods to transitioning solid established research results to a practical clinical setting and offer a framework for abbreviation disambiguation and co-reference resolution.
26.	Do BH, Wu AS, Maley J, Biswal S [26]	2013	The paper discusses developing an NLP system to extract fracture concepts from the text in real time, demonstrating automatic retrieval of bone fracture knowledge using natural language processing. This work developed and validated an NLP system. which extracts fracture and anatomy concepts from unstructured text and retrieves relevant bone fracture knowledge in real time and implements the system in an HTML5 web application to demonstrate a proof-of-concept feedback NLP system.
27.	Ho-Le TP, Center JR, Eisman JA, Nguyen TV, Nguyen HT [27]	2017	Artificial neural networks were trained and validated to predict hip fractures in post-menopausal women, achieving up to 87% accuracy, outperforming existing statistical models.
28.	Kolanu N, Brown AS, Beech A, Center JR, White CP [28]	2021	Natural language processing of radiology reports, like XRAIT, can efficiently identify patients with fractures, aiding in fracture detection and treatment prioritization in fracture liaison services.
29.	Wang Y, Mehrabi S, Sohn S, Atkinson EJ, Amin S, Liu H [29]	2019	The results verified the effectiveness of the proposed rule-based NLP algorithm in automatic identification of osteoporosis-related skeletal site-specific fractures from radiology reports and showed it could be utilized to accurately identify the patients with fractures and those who are also at high risk of future fractures due to osteoporosis.
30.	Lin Z, Dai W, Lai Q-Q, Wu H [30]	2023	In this article, a deep learning-based automatic detection algorithm was developed for rib fracture CT images of high-energy trauma patients, and the clinical effectiveness of this algorithm was evaluated.
31.	Bulstra AEJ, Buijze GA, Bulstra AEJ, et al. [31]	2022	An algorithm to guide non-orthopedic providers was developed after a review of the medical literature and fashioned with the aim of reducing complications and poor outcomes associated with delayed diagnosis of scaphoid fractures, affecting soldier health and unit readiness.
32.	Sharma S [32]	2023	AI in orthopedic X-rays shows potential to enhance fracture diagnosis accuracy and efficiency through advanced algorithms and integration with other imaging modalities, promising improved patient outcomes.
33.	Recht M, Bryan RN [33]	2017	AI, particularly machine learning, is viewed as a boon to radiologists, enhancing their value, efficiency, accuracy, and personal satisfaction, rather than posing a significant threat. These changes, particularly machine learning, will be a boon to radiologists by increasing their value, efficiency, accuracy, and personal satisfaction.
34.	Brink JA, Arenson RL, Grist TM, Lewin JS, Enzmann D [34]	2017	The future of radiology is heavily reliant on informatics and IT advancements, impacting decision support, big data correlation, data mining, and business analytics for improved resource utilization and decision-making. This article focuses primarily on areas where this IT transformation is likely to have a profound effect on the practice of radiology, including clinical decision support and business analytics.
35.	Oliveira E Carmo L, van den Merkhof A, Olczak J, et al. [35]	2021	In this article, external validation of a CNN on a temporally separate (separated by time) or geographically separate dataset is crucial to assess the generalizability of the CNN before application to clinical practice in other institutions.
36.	Rudolph, J., Schachtner, B., Fink, N. et al. [36]	2022	Clinically focused multi-cohort benchmarking is crucial for externally validating AI algorithm performance in basic chest radiography analysis, considering various clinical scenarios, reference standards, and expert comparisons.
37.	Goodman B, Flaxman S [37]	2017	It is argued that while deep learning will pose large challenges for the industry, it highlights opportunities for computer scientists to take the lead in designing algorithms and evaluation frameworks, which avoid discrimination and enable explanation.

## Conclusions

We hypothesize that AI could perform better than humans in many data-driven statistical activities. The biggest obstacles, though, will be resolving legal concerns and figuring out how to gather and interpret massive volumes of data effectively. Doctors will gain more from adopting AI than from rejecting it, even with its current drawbacks. Radiologists' roles are not confined to analyzing AI outcomes; neither do they substitute a professional clinician’s work. Instead, radiologists might employ AI techniques as an additional means to verify their uncertainties and choices. Radiologists must have a fundamental grasp of machine learning and tools that utilize AI to fully combine these fields; further study is required on the link between doctors and AI, particularly how to teach radiologists to utilize AI technologies and understand their findings. To enable more precise and quick diagnosis, AI systems need to keep adding medical applications to their repertoire.
